# Evolution of Plant Genome Size and Composition

**DOI:** 10.1093/gpbjnl/qzae078

**Published:** 2024-11-05

**Authors:** Bing He, Wanfei Liu, Jianyang Li, Siwei Xiong, Jing Jia, Qiang Lin, Hailin Liu, Peng Cui

**Affiliations:** Guangdong Laboratory for Lingnan Modern Agriculture, Genome Analysis Laboratory of the Ministry of Agriculture and Rural Area, Agricultural Genomics Institute at Shenzhen, Chinese Academy of Agricultural Sciences, Shenzhen 518120, China; Guangdong Laboratory for Lingnan Modern Agriculture, Genome Analysis Laboratory of the Ministry of Agriculture and Rural Area, Agricultural Genomics Institute at Shenzhen, Chinese Academy of Agricultural Sciences, Shenzhen 518120, China; Guangdong Laboratory for Lingnan Modern Agriculture, Genome Analysis Laboratory of the Ministry of Agriculture and Rural Area, Agricultural Genomics Institute at Shenzhen, Chinese Academy of Agricultural Sciences, Shenzhen 518120, China; College of Agronomy, Qingdao Agricultural University, Qingdao 266109, China; Guangdong Laboratory for Lingnan Modern Agriculture, Genome Analysis Laboratory of the Ministry of Agriculture and Rural Area, Agricultural Genomics Institute at Shenzhen, Chinese Academy of Agricultural Sciences, Shenzhen 518120, China; Guangdong Laboratory for Lingnan Modern Agriculture, Genome Analysis Laboratory of the Ministry of Agriculture and Rural Area, Agricultural Genomics Institute at Shenzhen, Chinese Academy of Agricultural Sciences, Shenzhen 518120, China; State Key Laboratory of Crop Stress Adaptation and Improvement, School of Life Sciences, Henan University, Kaifeng 475004, China; Guangdong Laboratory for Lingnan Modern Agriculture, Genome Analysis Laboratory of the Ministry of Agriculture and Rural Area, Agricultural Genomics Institute at Shenzhen, Chinese Academy of Agricultural Sciences, Shenzhen 518120, China; Guangdong Laboratory for Lingnan Modern Agriculture, Genome Analysis Laboratory of the Ministry of Agriculture and Rural Area, Agricultural Genomics Institute at Shenzhen, Chinese Academy of Agricultural Sciences, Shenzhen 518120, China; Guangdong Laboratory for Lingnan Modern Agriculture, Genome Analysis Laboratory of the Ministry of Agriculture and Rural Area, Agricultural Genomics Institute at Shenzhen, Chinese Academy of Agricultural Sciences, Shenzhen 518120, China

**Keywords:** Genome size, Polyploidy, Whole-genome duplication, Transposable element, GC content

## Abstract

The rapid development of sequencing technology has led to an explosion of plant genome data, opening up more opportunities for research in the field of comparative evolutionary analysis of plant genomes. In this review, we focus on changes in plant genome size and composition, examining the effects of polyploidy, whole-genome duplication, and alternations in transposable elements on plant genome architecture and evolution, respectively. In addition, to address gaps in the available information, we also collected and analyzed 234 representative plant genome data as a supplement. We aim to provide a comprehensive, up-to-date summary of information on plant genome architecture and evolution in this review.

## Introduction

The origin and early evolution of land plants date back to the mid-Paleozoic, between approximately 480 and 360 million years ago (MYA) [1]. The ∼ 350,000 extant land species exhibit remarkable diversity, including liverworts, hornworts, mosses, and vascular plants, with a wide range of complex organs and tissue systems. They are thought to have far-reaching consequences for the evolution of terrestrial organisms and the global environments [2]. The study of plant evolution has been conducted across several domains: the fossil record [3], morphology [4], and molecular approaches [5]. The development of genomic technologies has advanced the study of plant evolution, enhancing not only classification but also structural and functional exploration.

The first completed genome of *Arabidopsis thaliana* was published in 2000, with a total length of 115.4 Mb [6]. It is a small diploid genome and is regarded as a model system for structure and function studies of the plant genome due to its relatively simple genome content and growth cycle [6]. Whole-genome sequencing has made the complexity of the plant genome much clearer and more comprehensive. Through the comparative analyses of an increasing number of plant genome sequences and functional genomic datasets, we now have unprecedented insights into the impact of various factors on genome evolution. The purpose of this review is to provide an overview of our current understanding of plant genome size (GS) and composition. Based on our integrated data analysis and literature mining results, we focus on the variation in GS and composition among plants and the prevalence of polyploidy, and finally outline the impact of transposable elements (TEs) in plants.

## Genome size

**Figure 1:**
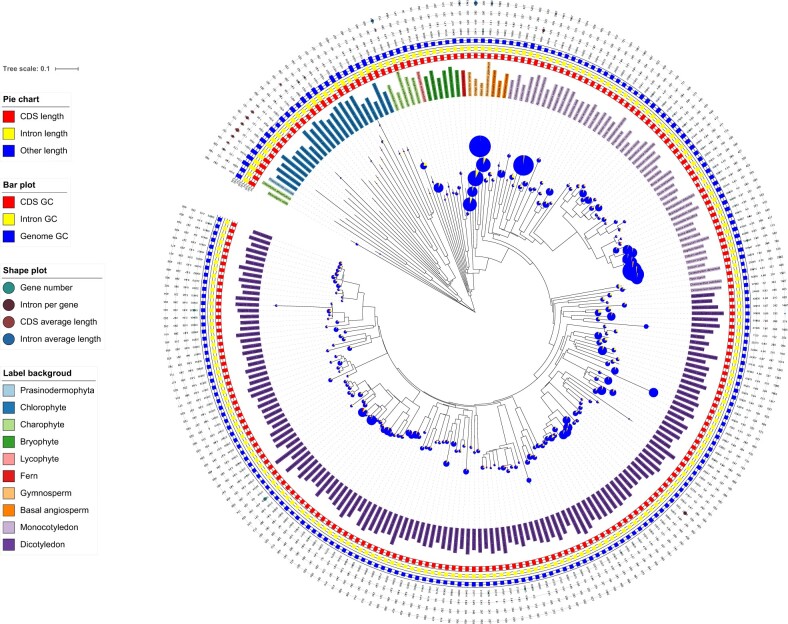
Global landscape of GSs across 234 representative plant species The pie chart illustrates GSs consisting of CDSs, introns, and others. The bar plot shows GC contents of the CDSs, introns, and genomes. The shape plot shows the number of genes, average intron number per gene, average CDS length, and average intron length. Different classifications are represented with different background colors in the “label region”. GS, genome size.

The total amount of DNA in the haploid or gametic nucleus of a species is referred to as the C-value or GS [7]. The GS of a species can be estimated using flow cytometry (FCM) [8] or by bioinformatic methods based on *k*-mer reads [9]. Among eukaryotes, GS varies approximately 64,000-fold [10], ranging from the smallest genome of the parasitic microsporidian *Encephalitozoon intestinalis* with a C-value of only 0.002 pg (2.3 Mb) [11] to the largest reliable GS estimated for the angiosperm *Paris japonica*, with a C-value of 152.23 pg (148,880 Mb) [12]. In plants, the Plant DNA C-DNA Database (https://cvalues.science.kew.org/) [13] indicates that GS can vary 2440-fold, similar to the 3300-fold variation observed in animals [14]. Among plants, angiosperms are particularly notable for their variability, with GS varying more than 2000-fold. Species with large genomes are largely restricted to monocots, especially in the families Alliaceae, Asparagaceae, Liliaceae, Melanthiaceae, and Orchidaceae [15]. Although the GSs of gymnosperms are extremely prominent among seed plants, their variation is more conservative, with GS ranging from 2.25 pg (*Gnetum ula*) to 36 pg (*Pinus ayacahuite*), reflecting an approximately 16-fold difference (**Figure 1**) [13]. Compared with seed plants, seed-free plants usually display smaller GS distributions [16]. Ferns and lycophytes, among seed-free plants, exhibit overall larger and more variable GSs than bryophytes. In contrast to ferns and lycophytes, there appears to be a correlation between GS and chromosome number in mosses and possibly in all bryophytes [16–18]. Furthermore, due to the extinction of many important ancestral species and the fact that a large number of plant genomes remain unquantified, further exploration of plant GS distribution patterns requires more analysis.

Consistent with the effects of polyploidization events, the expansion of plant GS enhances adaptation to specific environments [19]. Plant species with larger genomes typically have larger, sparser stomata, which limit stomatal conductance to CO_2_ and water [20] and slow the stomatal response to environmental changes [21]. The primary mechanisms promoting higher GS in plants are mainly polyploidization events, including whole-genome duplication (WGD), and the accumulation of substantial numbers of repetitive elements [16]. However, it is clear that the trend in GS is unlikely to be unidirectional, the so-called “one-way ticket to genome obesity” [22], but rather bidirectional, experiencing both contractions and expansions. The most typical example is *Arabidopsis*, which has undergone multiple WGD events [23]. If GS changes followed a unidirectional trend, *Arabidopsis* would currently be 48-ploid instead of diploid [24]. This conclusion is also supported by GS results in gymnosperms, where the range of GS fold change variation remains one of the smallest among plants, despite the prevalence of bulky genomes and the fact that the GS expansion mechanism differs from that of angiosperms in that TE insertion events rather than polyploidization events are the main cause of their amplification [25].

There are several explanations for the phenomenon of plant genome downsizing, such as the tendency to produce smaller genomes given the nitrogen (N) and phosphorus (P) loads of nucleic acids [26,27] or the selection for smaller genomes to improve photosynthetic and water use efficiency [19,28]. In terms of molecular mechanisms, GS contraction in angiosperms may be closely related to uneven and illegitimate recombination, as well as double-strand break (DSB) repair involving DNA recombination [16,29]. From the results of existing studies, it seems that there is a dynamic mechanism to regulate GS in plants. Under environmental stress or survival pressure, plants prefer to increase GS through polyploidization and TE insertion to improve their environmental adaptability. When the fitness landscape is stable, plants seem to exhibit genome downsizing to reduce the energy cost of large genomes [30]. It is noteworthy that, as one of the few relict species that survived the mass extinction, the generally large GS and narrow GS range of gymnosperms has raised questions about the ultimate destination of GS evolution in these species. It is interesting to consider whether these species have fully adapted to their current environment and are experiencing genome contraction, or if they still intend to pursue genome expansion.

## GC content variation

Typically, features such as gene size and GC content of the coding sequences (CDSs) have been shown to vary significantly both between different genomes and between different regions within the genome [31,32]. However, systematic studies in related areas have been scarce due to insufficient information on plant genomes. To explore this issue further, we obtained the genomes of 234 representative plant species with chromosome-level genome assemblies or unique evolutionary nodes from public databases. These plant species include 137 dicotyledons (22 in Fabales, 19 in Rosales, 12 in Solanales, 10 in Brassicales, nine in Malpighiales, and others), 51 monocotyledons (30 in Poales, five in Asparagales, four in Alismatales, three in Arecales, three in Dioscoreales, and others), three basal angiosperms, four gymnosperms, one fern, one lycophyte, six bryophytes, seven charophytes, 23 chlorophytes, and one prasinodermophyte (Table S1).

According to our results, the total gene size ranged from 9.88 Mb (74.84% of the GS) in chlorophytes to 710.04 Mb (7.43% of the GS) in gymnosperms. A high correlation coefficient was observed between gene size, intron size, and CDS size (gene size *vs*. intron size: *R* = 0.9565, *P* = 4.2E−126; gene size *vs*. CDS size: *R* = 0.5818, *P* = 1.37E−22). In many species of charophytes, gymnosperms, basal angiosperms, monocots, and dicots, the total intron size is more than half of the total gene size. Exon size appears to be limited by selective pressure (50 bp to 10 kb), whereas intron size varies widely within and between organisms (50 bp to 1 Mb). Most exon sizes in most species range from 100 bp to 1 kb, except for some species with broader exon sizes in chlorophytes and charophytes (*Chara braunii*). Intron size distributions tend to be unimodal (sharp or blunt) or bimodal (weak or a strong second peak) in plant species, and the size distribution patterns are highly variable among species. Further inspection reveals that intron size distributions are somewhat correlated with phylogeny, and species with closer evolutionary relationships have similar intron size distribution patterns. In general, according to our results, intron size distributions are relatively stable in dicots, although they are much more variable in other species, which is also consistent with previous conclusions [17,25].

**Figure 2:**
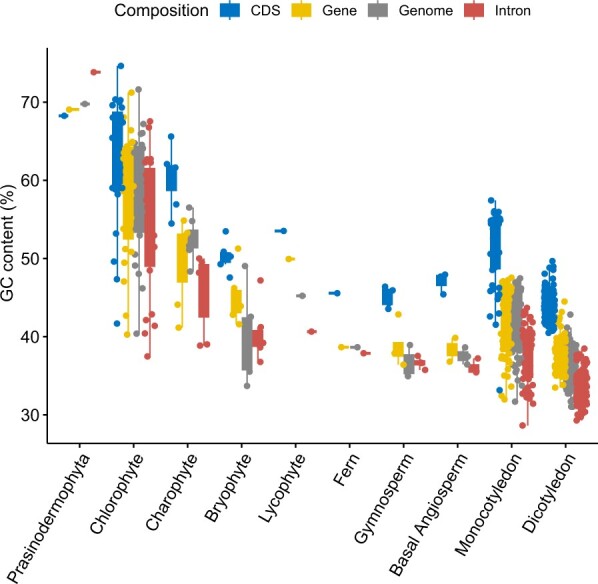
GC content results for different plant genome regions A total of 234 representative plant species were included in the GC content comparison, comprising 137 dicotyledons, 51 monocotyledons, three basal angiosperms, four gymnosperms, one fern, one lycophyte, six bryophytes, seven charophytes, 23 chlorophytes, and one prasinodermophyte.

From algae to higher plants, there is a trend of decreasing and then increasing GC content in plant genomes (**Figure 2**). *Raphidocelis subcapitata* in Chlorophyta has the highest genome GC content (71.62%), while *Cicer arietinum* in Dicotyledon has the lowest genome GC content (31.03%). Among them, plants in Chlorophyta have presented the highest diversity of genome GC content (from 40.38% to 71.62%, coefficient of variation = 0.1345). Compared with basal angiosperms, monocots and dicots have a higher diversity of genome GC content, and these observations identify the increase and decrease in the genome GC content of monocots and dicots. In addition, monocots have a higher average genome GC content than dicots (41.94% *vs*. 35.99%). Similar to the dynamics observed in the genome, the diversity of the GC content in genes, CDSs, and introns is similar. Most species have an increase in CDS GC content and a decrease in intron GC content compared with genome GC content, except for *Prasinoderma coloniale* in Prasinodermophyta and the genus *Ostreococcus* in Chlorophyta, which have higher intron GC content and lower CDS GC content. An increased GC content distribution for exon and intron is observed in Poaceae species, which is consistent with previous results [33,34].

**Figure 3:**
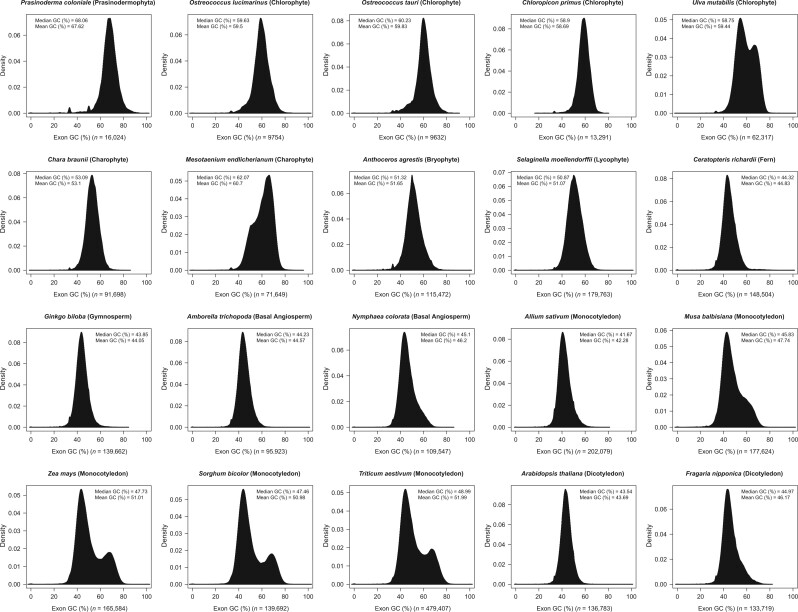
Distribution of GC content in the CDS region of representative plants CDS, coding sequence.

Several hypotheses have been proposed to explain the origin and evolution of changes in GC content, including selection for GC content, mutational bias, and GC-biased gene conversion (gBGC) [31,34–36]. gBGC is a recombination-related process that favors G/C over A/T bases during mismatch repair, and based on evidence for GC-biased enrichment of highly recombinant regions as well as GC-biased mismatch repair, it is increasingly argued that gBGC should be a major driver of changes in GC content [37–39]. Furthermore, the GC and GC3 contents of CDSs in dicotyledons show a unimodal distribution, whereas in monocot genomes, especially in grasses, they show a bimodal distribution. Interestingly, based on the results of our analysis, it seems that this phenomenon is not only present in monocots, but also in the green alga *Ulva mutabilis* with a bimodal distribution (**Figure 3**). It is worth noting that DNA sequence, as the ultimate carrier of genetic information, can be understood as a mapping of high-dimensional genetic information to lower dimensions. Therefore, highly compressed genomic features such as GC content may be the result of a combination of factors, and it is worth considering whether there is a highly robust mechanism involved.

## Plant polyploidy

Diploids reproduce primarily by producing haploid gametes, which combine to form diploid offspring. In rare cases, polyploidy can be produced either by somatic duplication of chromosomes in fertilized eggs or by the production of unreduced gametes, the latter being the primary mechanism of polyploidy production [40]. Polyploidy is more prevalent in plants than in animals, although its distribution among plants is not uniform [41]. For example, the frequency of polyploidy in ferns is as high as 95% [42], while in angiosperms the frequency ranges from 30% to 70%, with dicots generally exhibiting a higher degree of polyploidy than monocots [43]. On the other hand, natural polyploids are particularly rare in gymnosperms and are absent in cycads (Cycadophyta) or ginkgos (Ginkgophyta), although polyploid plants of ginkgos can be artificially cultivated under certain conditions [44,45]. The prevalence of polyploids in plants is intriguing because polyploids are anticipated to be rare and evolutionarily short-lived due to the frequency-dependent reproductive disadvantages associated with being the minority cytotype when they first arise in a population [46].

**Figure 4:**
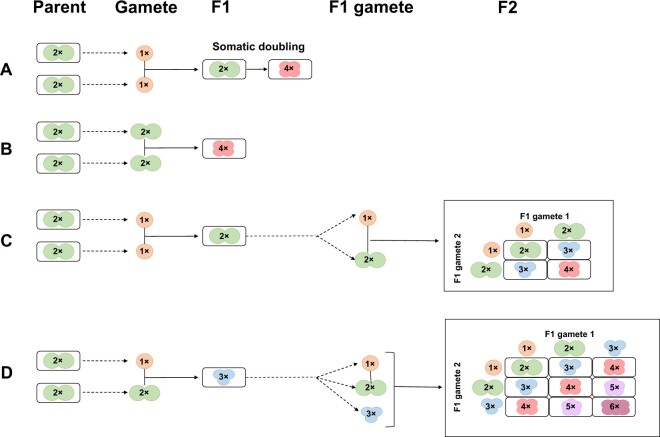
Different mechanisms of polyploid formation using tetraploid as an example **A**. Somatic cell doubling. **B**. Fusion of unreduced gametes. **C**. A certain percentage of aneuploid gametes may be produced in the F1 offspring. **D**. The triploid bridge scenario in which the intermediate triploid produces unreduced gametes, leading to polyploidy.

Although there are multiple causes of plant polyploid formation in terms of cytological pathways (**Figure 4**), most polyploid cultivars have been domesticated by wild-to-crop and interploidy introgression, and these two typical events are also important for the formation of allopolyploid and autopolyploid, respectively [47,48]. For example, hexaploid wheat (*Triticum aestivum*, AABBDD) is derived from a hybridization between cultivated tetraploid wheat (*T. turgidum*, AABB) and the goat grass *Aegilops tauschii* (DD) [49]. The agriculturally important genus *Brassica* contains three diploid species, *Brassica rapa* (AA, *n* = 10) [50], *B. nigra* (BB, *n* = 8) [51], *B. oleracea* (CC, *n* = 9) [52], and three allopolyploid species, *B. juncea* (AABB, *n* = 18) [53], *B. napus* (AACC, *n* = 19) [54], and *B. carinata* (BBCC, *n* = 17) [55]. Although previous studies have generally concluded that the number of autopolyploids is much lower than that of allopolyploids [56], ongoing studies suggest that the frequency and role of autopolyploidy in plants may be much higher [57,58].

Despite the large number of genes generated by polyploidy events that tend to be lost or non-functional, a growing body of evidence has shown that polyploidy plays a crucial role in enabling plants, especially angiosperms, in adapting to their environment and responding to stress, which may be an important driver of speciation [59–61]. In addition, polyploid species tend to show enhanced adaptations to salt stress [62], cold stress [63], or drought [64], and there appears to be a positive correlation between their frequency of distribution and latitude [65]. Although plants do not typically undergo polyploidization in stable environments, an excessively large genome can impose an additional energy burden on the organism. In times of high survival pressure, polyploidy events can lead to altered gene expression, epigenetic remodeling, or gene sub/neofunctionalization, providing a better buffer for plants [61]. It is worth noting that extant gymnosperms have also experienced several dramatic fluctuations in the Earth’s habitats over a long period of time, raising the question of whether the extreme rarity of polyploidy in gymnosperms implies that these species possess a fundamentally different mechanism for environmental adaptation compared with angiosperms.

The genome assembly of polyploids, especially homopolyploids, is challenging, because traditional diploid genome assembly strategies tend to ignore differences between haplotypes, resulting in chimeric haploid genome assembly, which can lead to significant errors for polyploids [66,67]. There are three main ideas to solve this problem: physical segregation methods, *de novo* haplotype assembly, and alignment-based phasing [68]. Although the physical segregation strategy is optimal for obtaining haplotypic gametophytes for sequencing, it has a significant human cost and presents several technical drawbacks [69]. The alignment-based polyploid phasing strategy, on the other hand, is based on well-assembled reference sequences and relies on sequence comparison, recording of phase-informative positions, optimization of objective functions [*e.g.*, minimum error correction (MEC)], and graph partitioning and clustering to construct haplotype genomes [70,71]. Alignment-based phasing has the advantage of being less computationally intensive, but the results obtained are highly dependent on the quality and structural complexity of the reference genome [72]. The strategy of *de novo* assembly is an attempt to reconstruct different haplotypes and resolve the structure of the genome using chromosomal long-range interactions (Hi-C) and/or parental information in the absence of reference sequences, and string graphs are also a common structure of existing algorithms under this strategy [73–76].

## WGD

WGD is a ubiquitous feature of plant genomes, contributing to variation in both GS and gene content. Although WGD has occurred in all major clades of terrestrial plants, its frequency is not fixed. More recent events have been observed in angiosperms, which are associated with increased rates of angiosperm diversification after the Cretaceous [77]. The two major clades of angiosperms, eudicots and monocots, both experienced paleopolyploidization events early in their evolutionary history, termed gamma (γ) and tau (τ), dating back to ∼ 120 MYA and ∼ 66 MYA, respectively [78,79]. However, the occurrence of recent WGD events in eudicots and monocots was completely independent. Two WGD events in monocots (ρ and σ) were inferred to have preceded the diversification of cereals and other grasses. And studies have revealed two recent WGDs (α and β) supported by *Arabidopsis* within the *Crucifer* lineage, as well as a triplication event (γ) likely shared by all core eudicots [78,80,81].

The ancestors of angiosperm lineages underwent a significant number of WGD events, as inferred from studies of gene duplication mutation patterns (Ks analysis), multi-tAxon paleopolyploidy search (MAPS analysis), and a combination of maximum likelihood and Bayesian approaches [82–84]. It should be noted that due to algorithmic or modeling differences, there may be variations in the estimation of differentiation time and speculation of WGD events among these methods. On the other hand, in gymnosperms, although studies based on transcriptomic data suggest that there was a common WGD event that occurred during the divergence of seed plants [85], this conclusion is controversial for angiosperms, and it is also intriguing whether there are lineage-specific WGD events in gymnosperms [86]. Recently, the nearly complete genome assemblies of *Ginkgo*, *Cycas*, and *Taxus* provided the syntenic genes to confirm a common seed plant WGD and no lineage-specific WGDs, putting these controversies to rest for the time being [25,87,88]. Compared with other land plants, the genomic characteristics of seed-free plants are significantly different, *e.g.*, hornworts, *Selaginella*, and most liverworts do not have WGD events and appear to have lower methylation levels [17]. In contrast, there has been at least one well-supported WGD in the history of most mosses, ferns, and homosporous lycophytes [89]. It is important to note that some key clades currently lack reference genomes, so further relevant data are needed for interpretation.

The main consequences behind WGD and polyploidy function involve the activation of TEs and epigenetic changes that subsequently lead to an increase in mutation rates and regulatory changes in genes and their genomic structure, ultimately leading to the diversification or the emergence of new phenotypes [77,90,91]. WGD is one of the major sources of genetic innovation in land plants, especially in angiosperms [90,92]. A commonly observed effect of WGD is the “gigas effect”, which refers to an increase in the size and robustness of plant traits [93]. The gigas effect is thought to be the result of polyploids having greater amounts of DNA, resulting in larger cells and translating into larger tissues and organs. There is evidence indicating that phenotypes may undergo deterioration or alteration in subsequent generations following WGD [94]. However, it is important to note that not all WGD events resulted in diversification. In particular, 46 out of 106 WGD events did not exhibit significant interactions with species formation in angiosperms [84].

## TEs in plants

**Figure 5:**
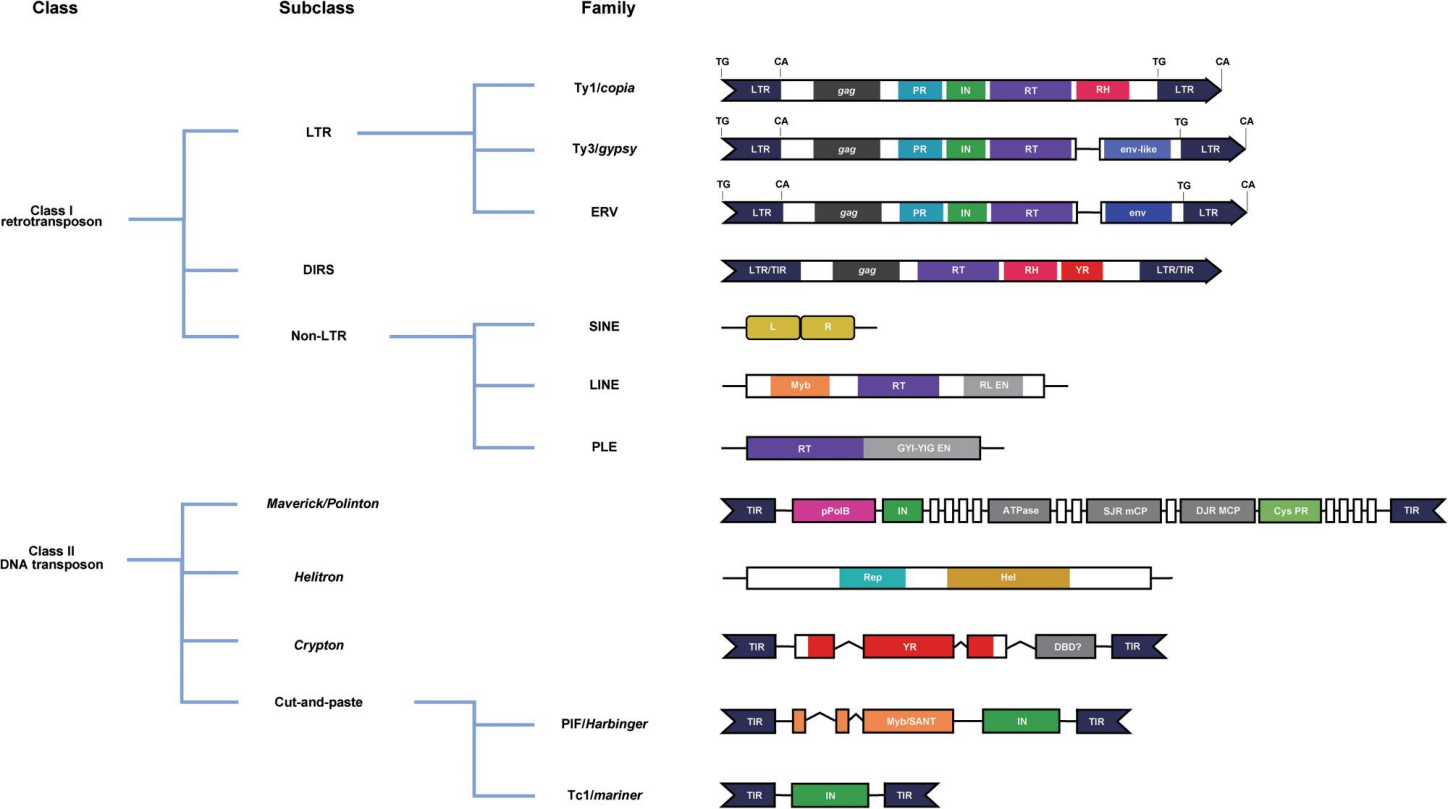
Classification and structural characteristics of major TEs in plants TE, transposable element; LTR, long terminal repeat; DIRS, *Dictyostelium* intermediate repetitive sequence; ERV, endogenous retrovirus; SINE, short interspaced nuclear element; LINE, long interspaced nuclear element; PLE, *Penelope*-like element; PIF, *P* instability factor; PR, protease; IN, integrase; RT, reverse transcriptase; RH, restriction endonuclease; YR, tyrosine recombinase; TIR, terminal inverted repeats; L, left; R, right; RL EN, RAG-like (recombination activating gene) enzyme; GYI-YIG EN, GYI-YIG endonuclease; SJR mCP, single jelly-roll minor capsid protein; DJR MCP, double jelly-roll major capsid protein; Cys PR, cysteine protease; Rep, replication protein; Hel, helicase; DBD, DNA binding domain.

TEs are mobile DNA sequences that can replicate independently within the host genome. Typically, they range in length from 100 bp to 10,000 bp [95]. Our understanding of the structure, organization, and translocation mechanisms of TEs has advanced considerably since their initial discovery in maize by Barbara McClintock [96]. Based on how these elements move through genomes, TEs can be divided into two main categories: retrotransposons (class I elements) and DNA transposons (class II elements). Retrotransposons use an encoded reverse transcriptase to synthesize DNA from RNA, increasing the copy number of the transposon. This mode of transposon movement is commonly referred to as “copy and paste”. DNA transposons utilize a “cut-and-paste” mechanism to jump from one locus to another within a genome (**Figure 5**) [97].

Class I retrotransposons can be further categorized into long terminal repeat-retrotransposons (LTR-RTs), non-LTR retrotransposons, and *Dictyostelium* intermediate repetitive sequences (DIRSs), with LTR-RTs being predominant in most plant species [98]. For example, LTR-RTs can comprise up to 80% of the maize genome [99]. LTR-RTs resemble retroviruses both in structure and mechanism and are flanked by conserved palindromic sequences (5′-TG … CA-3′) [100], ranging from a few hundred base pairs to more than 5 kb [101]. Most LTR-RTs contain two open reading frames (ORFs), *gag* and *pol*, where the *gag* ORF encodes the structural protein of the virus-like particle, while the *pol* ORF encodes aspartic proteinase (AP), reverse transcriptase (RT), RNase H (RH), and integrase (IN) [102]. New insertion of LTR-RTs creates a short repeat at the ends of the element, called a target site duplication (TSD), which is 4−6 bp long [103]. In addition, some plant LTR-RTs may contain additional open reading frames (aORFs), although their function is not yet clear [104]. Non-LTR retrotransposons are less abundant in plants, and can be divided into three major superfamilies: long interspaced nuclear elements (LINEs), short interspaced nuclear elements (SINEs), and *Penelope*-like elements (PLEs). Non-LTR retrotransposons lack LTRs and are transcribed from an internal promoter, and these elements can replicate themselves in the absence of the INT domain. The RT structural domain initiates DNA synthesis from the poly-A tail of the non-LTR retrotransposon transcript and ultimately ligates the newly synthesized DNA end to the insertion site [105].

Like class I transposons, class II transposons (DNA transposons) can be further subdivided into four subclasses: cut-and-paste, *Helitron*, *Crypton*, and *Maverick*/*Polinton* [106]. Cut-and-paste type DNA transposons, also known as terminal inverted repeat (TIR) TEs, tend to be the most abundant DNA transposons in plants and are characterized by autonomous copy-encoded transposases and terminally inverted repeats [107]. It is worth noting that these non-autonomous elements can retain binding sites recognized by autonomous transposons during transposition, resulting in an extensive MITE family that tends to have a greater ability to proliferate [108]. The most important feature of *Helitrons* is that they transpose with a “rolling cycle” mechanism via a transposase. This means that instead of a TIR, a strand of the original element is copied directly to the target site, a mechanism that is distinct from other DNA transposons [109]. *Cryptons* are relatively rare in eukaryotes and are also structurally similar to bacterial and archaeal insertion sequences [110]. *Maverick/Polintons* are widespread in eukaryotes. Although they are rare in number, they tend to be relatively large, often 15 kb or more in length. *Maverick/Polintons* are replicated by direct synthesis of DNA copies and are therefore known as self-synthesizing DNA transposons [95].

As with polyploidy events, the activity of TEs has played a major role in the evolution of plant genomes. Very few eukaryotic species are completely devoid of TEs [11], and most organisms that experience waves of TE mobilization also appear to contribute to the resizing and shaping of their genomes [111]. The average fraction of the genome occupied by TEs is ∼ 50% of the total genome, with a range from 10% to more than 85% [112]. In general, there is a negative correlation between GS and TE diversity in terrestrial plants. Typically, angiosperms tend to have smaller genomes than gymnosperms, but the TE landscape is more diverse, whereas most gymnosperms are dominated by LTR-RTs [95].

TEs have long been regarded as “junk DNA” and are typically masked during genome annotation to avoid influencing the annotation results. However, as research has advanced, it has become increasingly evident that TEs play an increasingly important role in both the structural and functional domains [113]. For example, TEs can enhance gene expression and produce phenotypic differences when integrated into the *cis*-regulatory region [114–116]. Additionally, they can function as “control elements” to mediate large-scale chromosomal rearrangements [117,118], together with extensive horizontal transfer [119]. Although TEs maintain an extremely high proportion in some plant genomes, their massive proliferation does not seem to be unconditionally tolerated by plants. As mentioned above, the enlarged genomes resulting from overactive TEs can increase the energetic burden on the organism.Available data suggest that DNA methylation mediated by the RNA-directed DNA methylation (RdDM) pathway and histone modification appear to be important mechanisms for regulating the expression of TEs [120–123]. Research on plant TEs remains in its early stages, encompassing the classification and identification of novel TE types as well as understanding their evolving roles in genome evolution and functional regulation, all of which require further investigation.

## Conclusion and future perspectives

As sequencing data continue to be published, plants have already shown great variation in GS, composition, and other important characteristics. Polyploidy and its associated WGD, along with the abundance of TEs, are the main reasons for the differences in their GSs. Based on the available results, plant adaptation and interactions with their living environments should be considered as significant drivers of plant genome evolution; this evolutionary process does not seem to be consistently stable and gradual. The significant differences in the genomic characteristics among various plants may indicate distinct strategies adopted by different plants to adapt to their respective environments. Conversely, these differences in the selection of environmental adaptation strategies may ultimately lead to divergent genomic landscapes. A typical example is the varying preferences for polyploidy and TEs between gymnosperms and angiosperms. To take another specific example, the average length of introns in gymnosperms is significantly greater than that in angiosperms. In cycads, for instance, the proportion of introns exceeds 50% of the genome, whereas in *Arabidopsis*, more than 90% of intron lengths are less than 1 kb [87]. Thus, whether in terms of energy loss through transcription and splicing or transcriptional regulation at three-dimensional or higher levels of gene organization, it is almost certain that genomic landscapes with such disparate characteristics will also have very different molecular regulatory mechanisms. In subsequent studies, it is essential to gather more genomic data from representative species, especially lower plants or key clades, to construct a complete evolutionary atlas of plant genomes. Additionally, the study of genome annotation and regulatory mechanisms in plants may require more attention.

## CRediT author statement

**Bing He:** Writing – original draft, Writing – review & editing. **Wanfei Liu:** Writing – original draft, Software, Formal analysis. **Jianyang Li:** Software, Formal analysis. **Siwei Xiong:** Software, Formal analysis. **Jing Jia**: Software, Formal analysis**. Qiang Lin:** Writing – review & editing, Investigation. **Hailin Liu:** Writing – original draft, Writing – review & editing, Validation. **Peng Cui:** Conceptualization, Writing – review & editing, Supervision. All authors have read and approved the final manuscript.

## Supplementary material

Supplementary material is available at *Genomics, Proteomics & Bioinformatics* online (https://doi.org/10.1093/gpbjnl/qzae078).

## Competing interests

The authors declare no competing interests.

## Supplementary Material

qzae078_Supplementary_Data
