# Supplementary material for: Evolution of Plant Genome Size and Composition
Source: Genomics Proteomics Bioinformatics. 2024 Nov 5;22(5):qzae078. doi: 10.1093/gpbjnl/qzae078 (PMC11630846; doi:10.1093/gpbjnl/qzae078)
Supplement: qzae078_Supplementary_Data [file qzae078_supplementary_data.zip › supplementary material captions.docx]

**Table S1 Summary of 234 representative plant genomes information**

This dataset includes species name, data link, publication, assembly, taxonomy, genome size, genome GC content, and others. In the publication column, we cited the corresponding paper of the genome with all the authors, publication title, journal, publication date, and Digital Object Identifier (DOI).
